# Optically pumped Milliwatt Whispering-Gallery microcavity laser

**DOI:** 10.1038/s41377-023-01264-6

**Published:** 2023-09-12

**Authors:** Huiqi Li, Zhaocong Wang, Lei Wang, Yang Tan, Feng Chen

**Affiliations:** School of Physics, State Key Laboratory of Crystal Materials, Shandong University, Jinan, China

**Keywords:** Solid-state lasers, Integrated optics

## Abstract

Whispering-gallery-mode microcavity lasers possess remarkable characteristics such as high *Q* factors and compact geometries, making them an essential element in the evolution of microlasers. However, solid-state whispering-gallery-mode lasers have previously suffered from low output power and limited optical conversion efficiency, hindering their applications. Here, we present the achievement of milliwatt laser emissions at a wavelength of 1.06 µm from a solid-state whispering-gallery-mode laser. To accomplish this, we construct a whispering-gallery-mode microcavity (with a diameter of 30 µm) using a crystalline Nd: YAG thin film obtained through carbon-implantation enhanced etching of a Nd: YAG crystal. This microcavity laser demonstrates a maximum output power of 1.12 mW and an optical conversion efficiency of 12.4%. Moreover, our unique eccentric microcavity design enables efficient coupling of free-space pump light, facilitating integration with a waveguide. This integration allowed for single-wavelength laser emission from the waveguide, achieving an output power of 0.5 mW and an optical conversion efficiency of 6.18%. Our work opens up new possibilities for advancing solid-state whispering-gallery-mode lasers, providing a viable option for compact photonic sources.

## Abstract (web summary)

We achieve milliwatt laser emissions at 1.06 µm wavelength from a 30 µm solid-state whispering-gallery-mode laser, constructed using Nd:YAG thin films obtained through carbon-implantation enhanced etching of a Nd:YAG crystal.

## Graphical abstract

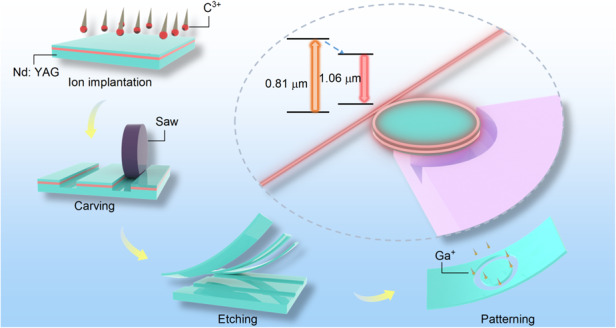

## Introduction

Whispering gallery mode (WGM) microlasers have undergone remarkable advancements in the past decade and have become indispensable in the evolution of laser technology^1–5^. The WGM configuration boasts exceptional optical characteristics, such as high-quality factors^6–9^ and small mode volumes, ensuring low lasing thresholds^10^ and extraordinary sensitivity to the surrounding environment^11–13^. Depending on the lasing mechanism, lasers can be either semiconductor (typically electrically pumped)^10,14^ or solid-state (optically pumped) lasers^15–18^. Semiconductor WGM lasers enable milliwatt laser emissions^19,20^ and compact, integrated designs based on silicon or III–V-based platforms^21–24^. These lasers leverage the inherent advantages of semiconductors, including well-established manufacturing processes and high refractive indices, which have been widely acknowledged as the primary avenue for developing on-chip laser sources for photonic integrated circuits. On the other hand, solid-state WGM lasers, as an alternative lasing strategy, have garnered continuous and intense interest^25–27^ since the first observation of optical WGM in 1961^28^. Their unique three/four-level lasing system has found broad applications not only in on-chip light sources but also in non-Hermitian optics^29,30^, optical communication^31^, biosensors^32,33^, and more. However, unlike semiconductor WGM lasers that can achieve milliwatt laser emissions, solid-state WGM lasers suffer from much lower output powers (typically only a few microwatts) and optical conversion efficiencies (typically less than 1%) (Table S1). Recently, a microring laser based on titanium-doped sapphire (Ti: Sa) has been reported to exhibit a laser emission of 0.5 mW, but it comes with a high threshold (6 mW) and low optical conversion efficiency (~0.5%)^34^. These inadequate laser performances of solid-state WGM lasers severely limit their applications in photonics.

One critical factor contributing to the underperformance is using a suboptimal gain medium. Commonly used gain mediums for solid-state WGM lasers, such as rare-earth-doped lithium niobate (LiNbO_3_ or LN) and silica, suffer from low emission/absorption cross-sections and relatively poor thermal stability, making them less than ideal choices. Therefore, there is a strong desire to explore alternative gain mediums for on-chip WGM microlasers. An excellent option is the rare-earth-doped yttrium aluminum garnet (YAG) crystal^35–39^, which is widely recognized as the most popular and successful lasing medium in well-established solid-state bulk lasers. YAG crystal can be doped with various rare-earth elements, allowing for laser emission across a broad range of wavelengths, including ~2.9 μm (Er)^40^, 2 μm (Tm)^41^, 1.6–1.4 μm (Cr)^42^, 1.3 μm (Nd)^43^, 1.06 μm (Nd)^44^, and 1.03 μm (Yb)^45^. Notably, the Nd: YAG crystal, with its four-level laser system, exhibits a remarkably low lasing threshold and is well-suited for on-chip applications, nonlinear optics, and biosensors, which has been extensively discussed as an on-chip light source based on waveguides^46–48^. The main challenge to its application as WGM microlaser is to extract the membrane structure from the doped YAG bulk crystal.

Another potential obstacle in implementing on-chip solid-state WGM lasers lies in the pumping method. The most desired approach is integrating semiconductor lasers onto the same photonic platform as the rare-earth-doped solid-state laser, allowing for electric pumping and compactness at the system level^34^. However, due to the challenges involved in heterogeneous integration on the semiconductor platform, the standard pumping method is to couple the pumping laser from an off-chip light source into the waveguide^15,16^, fiber taper^17,18,26^, or prism^27^, and then into the WGM, which result in a low efficiency of energy injection into the microcavity. Therefore, there is a strong motivation to develop a novel coupling technique that enables direct injection of the pumping laser into the on-chip WGM.

Here, we have fabricated an ultrathin Nd: YAG film by peeling it off from the bulky Nd: YAG crystal, and then shaping it into a microcavity. We excite these Nd: YAG microcavity lasers through fiber-taper coupling, resulting in milliwatts laser emission. To further realize the free-space coupling of the pumping laser, we have introduced an air hole with a diameter of 4 µm onto the microcavity, creating an eccentric microcavity^49^. The eccentric microcavity provides a novel approach to excite the microcavity for a high-power on-chip light source.

## Results

**Fig. 1:**
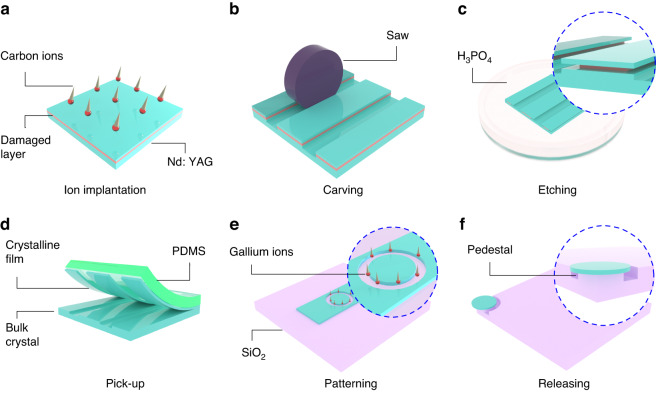
Preparation of crystalline microcavity. **a** Nd: YAG crystal irradiated by carbon ions. **b** Grooves diced by a wafer saw to expose the damaged layer (red layer). **c** Damaged layers corroded by phosphoric acid. **d** Crystalline films picked up from the Nd: YAG substrate by PDMS. **e** Microcavity fabricated by FIB milling. **f** Microcavity on the pedestal

**Fig. 2:**
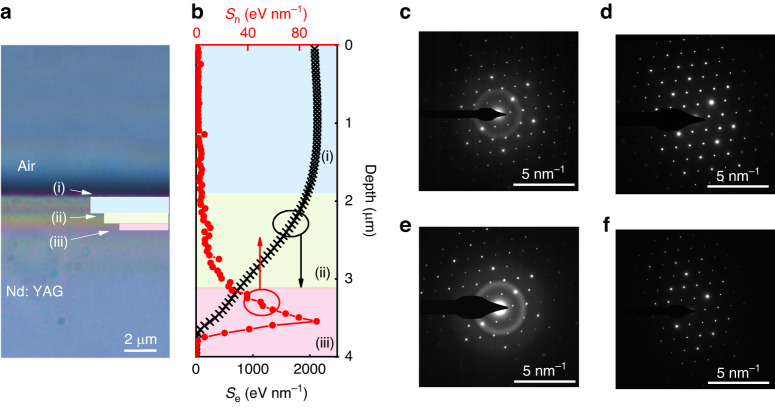
Modification of Nd: YAG via ion irradiation. **a** Photograph image of the irradiated Nd: YAG crystal. Scale bar 2 μm. **b** Distributions of electronic energy loss (*S*_*e*_), marked as black cross, and nuclear energy loss (*S*_*n*_), marked as red dot, versus the injection depth calculated by SRIM-2018. **c**–**e** display the SAED (selected area electron diffraction) image of Nd: YAG with different depths, marked as (i), (ii) and (iii) in **b**. Scale bar 5 nm^−^^1^. **f** Display the SAED image of the exfoliated Nd: YAG film. Scale bar 5 nm^−1^

Figure 1 illuminates preparation procedures employed in creating the free-standing Nd: YAG film and microcavity. The Nd: YAG crystal with a dimension of 10 × 10 × 1 mm^3^ has one biggest facet with optical polishing. The polished facet is implanted by the carbon (*C*^3+^) ions at an energy of 6 MeV and fluence of 2 × 10^15^ ions cm^−2^ (Fig. 1a). The incident direction of the carbon beam is at an angle of 7° off the normal direction of the facet to avoid the channeling effect. Then the implanted facet is diced into grooves (with a depth of 10 µm) with a separation of 100 µm by a wafer saw (DISCO Co., P1A851-SD4000-R10-B01) to expose the damaged layer (Fig. 1b). Afterward, the sample is immersed in the phosphoric acid (80%) at 80 °C for 12 h. The acid corrodes the damaged layer and exfoliates a thin crystalline film (thickness of 1 µm) from the bulk crystal (Fig. 1c). The Supplementary Material includes a video demonstrating the corrosion process. To pick up the exfoliated film, we employ the mechanical transfer method^50–52^ with Polydimethylsiloxane (PDMS), as depicted in Fig. 1d. Figure S2 displays Atomic Force Microscope (AFM) images of the exfoliated film. The top facet (the boundary of Sections I and II in Fig. 2) exhibits a roughness of 0.574 nm rms (Figure S2a), while the bottom facet (the boundary of Sections II and III in Fig. 2) has a roughness of 0.729 nm rms (Figure S2b). Notably, both sides have roughness levels in the sub-nanometer range, comparable to a polished surface^53^, ensuring minimal scattering loss within the microcavity. Using focused ion beam (FIB) milling, we pattern the free-standing Nd: YAG 1 µm film into a microcavity with a diameter of 30 µm (Fig. 1e, Figure S3). Finally, the microcavity is transferred to a pedestal using the PDMS-assisted site-specific transfer method (Fig. 1f).

Figure 2a shows the photograph of the irradiated Nd: YAG cross-section, revealing distinct stratification near the surface that corresponds to different crystalline conditions. During implantation, incident *C*^3+^ ions interact with the atomic nucleus and electrons in the Nd: YAG crystal through elastic and inelastic scattering, respectively. This leads to nuclear (*S*_n_) and electronic (*S*_e_) energy losses, decreasing the crystalline quality of the Nd: YAG crystal. In Fig. 2b, the distribution of electronic (nuclear) energy loss as a function of injection depth is presented, which is simulated using the SRIM-2018 module (stopping and range of ions in matter). The incident *C*^3+^ ions penetrate the Nd: YAG crystal, generating electronic energy loss near the surface, labeled as (i) in Fig. 2b. Finally, the *C*^3+^ ions stop inside the Nd: YAG crystal, creating a buried damaged layer at the thickness of 900 nm^54–56^. This layer is located at a depth of 3.5 µm from the surface, as shown in Fig. 2b. Between regions (i) and (iii), the damage is relatively minor, as indicated by region (ii) in Fig. 2b. Figure 2c–e and Fig. S1a–c display Selected Area Electron Diffraction (SAED) and Transmission Electron Microscopy (TEM) images of regions (i), (ii), and (iii), respectively. In Fig. 2d, e, obvious amorphous halos can be observed, indicating that the lattice structure of regions (i) and (iii) has been damaged during ion irradiation. However, the amorphous halos have disappeared in Fig. 2d, suggesting that ion irradiation has caused little damage in (ii) layer. Then the irradiated Nd: YAG crystal is immersed in phosphoric acid at 80 °C for 12 h. Regions (i) and (iii) are completely corroded, leaving only region (ii) with a thickness of 1 µm. Figure 2f (Fig. S1d) shows the SAED (TEM) images of the exfoliated region (ii), indicating that the crystal structure of the exfoliated film remains intact.

**Fig. 3:**
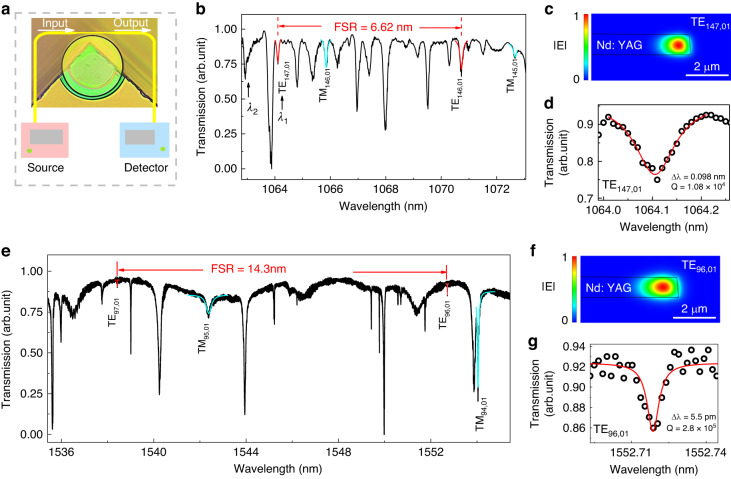
Analysis of resonant modes. **a** Schematic of the experimental setup; **b** Transmission spectrum of Nd: YAG microcavity in the laser emission band, with FSR (free spectral range) of 6.62 nm. Simulated Cross section of absolute of the electric field distribution **c** and the transmission spectrum **d** of TE_147,01_, with *Q* factor of 1.08 × 10^4^. **e** Transmission spectrum of the Nd: YAG microcavity in the telecom wavelengths, with FSR (free spectral range) of 14.3 nm. Simulated Cross-section of absolute of the electric field distribution **f** and the transmission spectrum **g** of TE_96,01_, with *Q* factor of 2.8 × 10^5^

We measure the transmission spectrum of the Nd: YAG microcavity in the laser emission band (from 1060 to 1075 nm wavelength) of Nd: YAG crystal, following the experimental setup displayed in Fig. 3a. To ensure accurate results and avoid interference from thermal effects, we keep the power of the probe light at a low level. To determine the free spectral range (*FSR*), we identify resonant modes in the transmission spectrum via the finite element method (COMSOL Multiphysics) utilizing the strategy described in Ref. ^57^. This Nd: YAG microcavity, with a thickness of 1 μm, supports both transverse electric (TE) and magnetic (TM)—like modes. These modes are classified by their azimuthal and radial orders, denoted as TE_m,n_ (or TM_m,n_), respectively, where *m*, *n* are the azimuthal and radial mode numbers. Based on the calculated results, we have labeled the lowest radial order of TE (TE_147,01_, TE_148,01_) and TM (TM_146,01_ and TM_145,01_) modes in Fig. 3b. The *FSR*, determined by the spectral range between the lowest radial modes (TE_147,01_ and TE_148,01_), is found to be 6.62 nm. This value is consistent with the theoretical result (6.626 nm) calculated by ${FSR}\approx \frac{{({\lambda }_{i})}^{2}}{2\pi R{n}_{i}}$ (*i* is an integer; *λ*_*i*_ is the *i*-order resonant wavelength; *R* is the radius of the microcavity; *n*_*i*_ is the effective refractive index of the resonant wavelength).

Figure 3c, d show the simulated cross-sectional mode profile and the transmission spectrum of TE_147,01_, respectively. The *Q* factor for TE_147,01_ is determined to be 1.08 × 10^4^ through Lorentzian fitting. The measured *Q* factor of the Nd: YAG microcavity is influenced by the Nd-ion absorption, intrinsic radiative (curvature) loss, and scattering losses of the microcavity, as the Nd: YAG crystal has optical absorption in this detection band. To eliminate the impact of the absorption of the doped Nd ions, we measure the transmission spectrum (Fig. 3e) of the Nd: YAG microcavity in the telecom wavelengths (1535–1558 nm), which is outside the absorption range of the doped Nd ions. Figure 3f, g show the simulated cross-sectional mode and the high-resolution transmission spectrum, respectively, corresponding to the lowest radial TE mode (TE_96,01_) in the telecom wavelength range. The estimated *Q* factor for this mode is 2.8 × 10^5^.

**Fig. 4:**
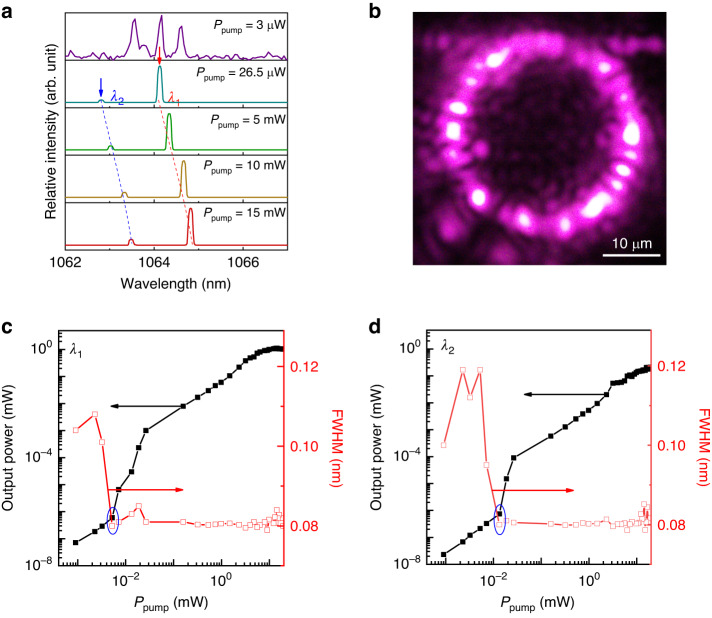
Laser performance of Nd: YAG microcavity. **a** Laser spectra at ~1060 nm pumped by 810 nm laser with different pumping power, here *λ*_1_ refers to the laser mode marked with a red arrow, *λ*_2_ refers to the laser mode marked with a blue arrow. **b** The optical microscope image of the emission from Nd: YAG microcavity. Scale bar, 10 μm. Output laser power (black dot) and spectral width (red dot) versus pump power for *λ*_1_ **c** and *λ*_2_ **d**, respectively

The laser emission from the Nd: YAG microcavity is excited by a continuous-wave (CW) pumping laser with a central wavelength of 810 nm through fiber-taper coupling. Figure 4a illustrates the changes in the output signal spectra as the pumping power (*P*_pump_) increases. In this study, *P*_pump_ refers to the power of the pumping laser coupled from the fiber taper into the microcavity. The coupling efficiency between the fiber taper and the Nd: YAG microcavity is 40%. At *P*_pump_ = 3 µW, only fluorescence with a broad spectral width and low intensity is observed. When *P*_pump_ is increased to 26.5 µW, laser emission is observed at two different wavelengths: 1064.12 nm (*λ*_1_) and 1062.82 nm (*λ*_2_). These wavelengths correspond to the TE_147,01_ and TM_132,03_ modes, respectively. Continuously increasing *P*_pump_ to 15 mW results in a redshift of the *λ*_1_ (*λ*_2_) laser by ∆*λ* = 0.7 nm (0.66 nm). This redshift is supposed to be caused by the thermal effect of the microcavity. Figure 4b shows a microscope photograph of the WGM microcavity under *P*_pump_ of 15 mW, with the pumping laser filtered by a mirror (see Materials and Methods section). The photograph clearly shows the laser oscillations in the microcavity and output lasers in the fiber along the forward and reverse directions.

Figure 4c, d illustrate the variations in the characteristics of *λ*_1_ and *λ*_2_ lasers related to *P*_pump_. The relationship between output power and pumping power is plotted on a log-log scale, and both *λ*_1_ and *λ*_2_ exhibit similar trends. For *P*_pump_ values greater than 5 (13) µW, the output power of the *λ*_1_ (*λ*_2_) laser increases rapidly. However, this increase slows down slightly when *P*_pump_ exceeds 26 (26) µW, as the thermal effect of the microcavity begins to impact the resonance mode and the fluorescence of the Nd: YAG crystal. Once the *P*_pump_ exceeds 5.5 (5.5) mW, the output power of the *λ*_1_ (*λ*_2_) laser reach saturation. Furthermore, the linewidth of the *λ*_1_ (*λ*_2_) laser exhibits a similar trend, narrowing significantly from approximately 0.1 nm to 0.08 nm at *P*_pump_ = 5 (13) µW and fluctuating for *P*_pump_ values greater than 5.5 (5.5) mW. This indicates that the *λ*_1_ (*λ*_2_) laser has a threshold of 5 (13) µW, and the Nd: YAG microcavity reaches a state of gain saturation when *P*_pump_ exceeds 5.5 (5.5) µW. Besides *λ*_1_ (*λ*_2_) laser has a maximum optical conversion efficiency of 12.4 (1.29)% and a maximum output power of 1.12 (0.20) mW.

**Fig. 5:**
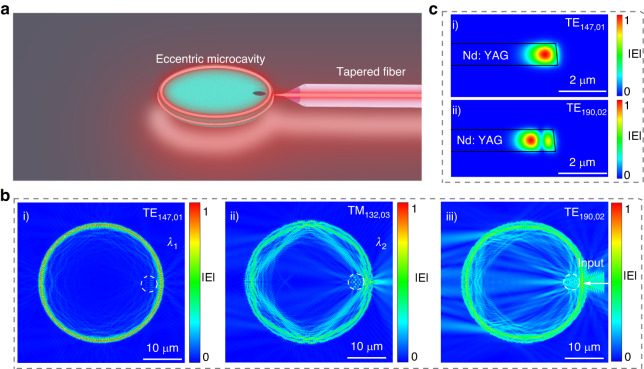
Design of eccentric microcavity. **a** Schematic diagram of the free-space coupling of the eccentric microcavity. **b** Cross-section of absolute of the electric field distribution of (i) TE_147,01_ corresponding to *λ*_1_ (ii) TM_132,03_ corresponding to *λ*_2_ (iii) TE_190,02_ corresponding to the pumping laser. Scale bar, 10 μm. **c** Simulated absolute of the electric field distribution of (i) TE_147,01_ corresponding to *λ*_1_, (ii) TE_190,02_ corresponding to the pumping laser at 810 nm. Scale bar, 2 μm

In order to achieve the free-space coupling of the pumping laser, we design a suitably positioned air hole in the Nd: YAG microcavity, so-called eccentric microcavity. As shown in Fig. 5a, the pumping light (at 810 nm) enters the eccentric microcavity through a tapered fiber, aligned with the axis of the microcavity and the center of the air hole. The air hole, with a low refractive index, acts as a concave lens, effectively coupling and scattering the input light into the high-order radial mode (*n* > 1) of the microcavity. Meanwhile, the air hole does not interfere with the fundamental radial mode (*n* = 1) of the microcavity, thanks to the careful selection of the distance between the boundaries of the hole and the microcavity. To determine the optimal parameters for the air hole, we have conducted a set of numerical simulations on the eccentric microcavity, considering different positions (*d*) and radii (*r*) for the air hole. As shown in Figure S4, the optimal values for *d* and *r* are 11.2 µm and 2 µm, respectively. These values ensure optimized free-space coupling efficiency for the high-order radial mode of the 810 nm laser, while minimizing scattering losses for the TE_147,01_ mode of the ~1060 nm laser.

We conduct simulations to investigate the light propagation in an eccentric microcavity (with *r* = 2 µm and *d* = 11.2 µm) in order to analyze the contribution of the hole to the distribution of light intensity (Fig. 5b). In Fig. 5bi, we observe the TE_147,01_ mode corresponding to the *λ*_1_ laser in the microcavity, which exhibits stable resonance. In Fig. 5bii, we introduce the high-order radial mode TM_132,03_ associated with the *λ*_2_ laser into the eccentric microcavity. The presence of the hole induces scattering losses for the TM_132,03_ mode, resulting in an increased loss for higher-order radial modes (*n* > 1). This additional loss helps to suppress the excitation of multiple laser modes and enables the realization of single-mode laser emission.

Figure 5biii displays the absolute electric field distribution of the pumping light at 810 nm. In this case, the hole scatters the pumping light, generating a high-order radial mode with concentrated intensity in TE_190,02_. Furthermore, Fig. 5c illustrates the overlapping of TE_190,02_ at 810 nm with TE_147,01_ at 1064.7 nm. To quantify this overlap, we utilize the following equation^58^ to determine the modal overlap factor.

1 $$\zeta =\frac{\int {E}_{1}{E}_{2}{dxdy}}{\sqrt{\int {\left|{E}_{1}\right|}^{2}{dxdy}}\sqrt{\int {\left|{E}_{2}\right|}^{2}{dxdy}}}$$

where *E*_i_ (*i* = 1, 2) are the electric fields of TE_190,02_ at 810 nm, and TE_147,01_ at 1064.7 nm modes, respectively. The TE_190,02_ of the pumping laser has a distinctive spatial symmetry, leading to a degraded spatial mode overlap with TE_147,01_ of the exciting laser. According to Eq. (1), *ζ* for the free-space coupled eccentric microcavity is calculated to be 86.12%. In the case of the fiber-taper-coupled microcavity, both the exciting (TE_147,01_) and pumping (TE_198,01_) lasers are in the fundamental radial mode (*n* = 1), and their *ζ* is determined to be 98.31% based on the same equation. While the *ζ* value is smaller in the free-space coupled eccentric microcavity (86.12%), it is still sufficient for laser oscillation and achieves a high laser conversion efficiency compared to fiber-taper coupling.

**Fig. 6:**
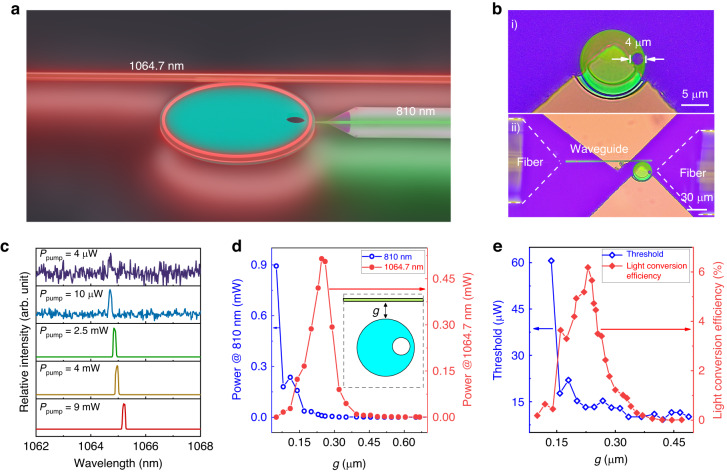
Laser performance of eccentric microcavity. **a** Schematic diagram of eccentric microcavity coupled with a waveguide. **b** (i) Photograph of the eccentric Nd: YAG microcavity, Scale bar, 10 µm. (ii) The photograph of the free-space coupling setup for the laser emission from the waveguide. Scale bar, 30 µm. **c** Spectra of the output laser from the waveguide with the pumping power arranging from 0.004 mW to 9 mW. **d** Power of the light coupled into the waveguide at 810 nm (1064 nm) versus the relative distance of waveguide and the microcavity. **e** The optical conversion efficiency and the threshold of the output laser as a function of the relative distance

Figure 6a displays the experimental investigation on the laser emission of the eccentric Nd: YAG microcavity. An undoped YAG waveguide (Figure S3d) is used as the output port, while the eccentric Nd: YAG microcavity is excited by a free-space coupled 810 nm laser. The eccentric microcavity had dimensions of *R* = 15 µm, *r* = 2 µm, and *d* = 11.2 µm (Fig. 6bi). The experimental setup, depicted in Fig. 6bii, involves the waveguide and Nd: YAG microcavity on two separate mobile stages. The gap width (*g*) between the waveguide and microcavity is adjusted to control their coupling efficiency. The pumping laser is directly coupled to the eccentric microcavity using a tapered fiber with a coupling efficiency of 10%, and the output laser from the waveguide is collected using another tapered fiber. Figure 6c shows the evolution of the output spectra at different pumping powers, with *g* fixed at 0.23 µm. At pumping powers less than 10 µW, only a broad emission gain background is observed. However, increasing the pumping power to 10 µW resulted in a laser signal with a narrowed linewidth (0.08 nm) at 1064.7 nm. Unlike the Nd: YAG microcavity (Fig. 4a), the eccentric microcavity has a single wavelength output due to the air hole, which introduces high loss with *n* > 1 (Fig. 5bii). Further increasing the pumping power led to a red shift (∆*λ* = 0.52 nm) in the output spectra due to the heating effect of the microcavity. Figure 6d illustrates the light power coupled into the waveguide for different *g* values, with a constant pumping power of 9 mW. When *g* is higher than 0.23 µm, the waveguide emits laser at ~1.06 μm, and the power increases as *g* decreases. Conversely, for *g* values lower than 0.23 µm, the pumping laser at 810 nm is also observed in the waveguide, and its power exponentially increased with decreasing *g*. Simultaneously, the power of the ~1.06 μm laser sharply decreased as *g* decreased.

Figure 6e illustrates the relationship between the optical conversion efficiency and the threshold as g varies. At *g* = 0.23 µm, the optical conversion efficiency reaches a maximum of 6.18%. For *g* longer than 0.23 µm, the laser threshold of the eccentric microcavity remains below 15 µW, but it quickly rises to 60 µW when *g* is reduced to 0.136 µm. The eccentric microcavity changes laser performance at *g* = 0.23 µm, which is attributed to insufficient absorption of the pumping light due to the guidance into the waveguide. Compared to the microcavity laser shown in Fig. 4, the eccentric microcavity laser has a lower optical conversion efficiency due to its smaller *ζ*. The eccentric configuration offers a flexible integration strategy with the waveguide, allowing for constant input pumping through free-space coupling without the need for initial coupling of the pumping laser into the waveguide. The exciting laser can be introduced into the integrated photonics chip by placing the microcavity close to the waveguide.

## Discussion

Our work demonstrates the generation of milliwatt laser emission using a WGM solid-state laser that utilizes the Nd: YAG crystal. To achieve this, we propose the carbon-implantation enhanced etching as an efficient approach to exfoliate a crystalline film from Nd: YAG crystal. The surface of the Nd: YAG film exhibits exceptional smoothness, providing a solid foundation for fabricating optical components at the micron scale. Subsequently, we pattern the Nd: YAG film into a free-standing WGM microcavity with a diameter of 30 µm. The WGM laser, based on this Nd: YAG microcavity, achieves a maximum laser emission of 1.12 mW and an optical conversion efficiency of 12.4%. These values are 2 orders higher than those reported for rare-earth doped LN microcavities (Supplementary IV).

The WGM laser, presented in this study, is a solid-state laser that offers a parallel laser approach distinct from semiconductor lasers (or diode lasers). While direct electrical pumping of semiconductor lasers is a significant trend for on-chip light sources, diode lasers alone cannot meet all the requirements of photonic platforms. Therefore, solid-state lasers with different lasing mechanisms are an alternative option for photonic platforms. Similar to the widely used off-chip diode laser-pumped solid-state lasers, we believe that integrating diode lasers as the pumping source for solid-state lasers can provide on-chip light sources more flexibility. Our work proposes a novel technology combining a free-standing YAG crystal with a waveguide, resulting in a hybrid structure.

Furthermore, the YAG crystal can be doped with various rare earth elements, including Tm, Cr, Yb, Nd, and Er, resulting in a wide range of laser emissions at wavelengths ranging from 2.9 to 1.03 *μ*m. Our research introduces a novel technique for extracting microdisks from bulk YAG crystals, applicable to all types of doped YAG crystals. To illustrate this technique, we focus on the example of Nd: YAG microdisks. We believe that our work will significantly contribute to advancing hybrid-integrated photonics.

## Materials and methods

### Fabrication of Nd: YAG microcavity

The polished Nd: YAG crystal was implanted with carbon ions with the energy of 6 MeV and fluence of 2 × 10^15^ ions cm^−2^ and then annealed at 400 °C to repair unnecessary damage. The surface was then cut to expose the damaged layer. After immersing the crystal in phosphoric acid solution at 80 °C for 12 h, the Nd: YAG crystalline films with a thickness of 1 μm were exfoliated. The crystalline films were transferred to silica substrate by PDMS for post-processing. The microcavities and the eccentric microcavity were fabricated by FIB milling and then transferred on a pedestal by PDMS.

### Optical characterization

In order to characterize the microcavity, a tunable laser was used as the probe light, inputted into a fiber taper, and coupled into the microcavity. The transmission spectrum was detected by a photoelectric detector and displayed on an oscilloscope. In order to characterize the laser operation of the microcavity, an semiconductor laser with a central wavelength of 810 nm was connected to tapered fiber as a pump laser and then coupled into the microcavity, and the emission laser was observed by a spectrometer. When detecting the laser operation of the eccentric microcavity, we placed the Nd: YAG eccentric microcavity and the un-doped YAG waveguide onto two stages and used a set of tapered fibers as the input and the receiving end. The coupling distance between the microcavity and the waveguide was precisely controlled by an electric motorized stage with an accuracy of 10 nm.

### Numerical simulation

The absolute distribution of electric field distribution in Fig. 5bi–iii were calculated by the FDTD solution. The boundary condition was the perfect matching layer (PML). For Fig. 5bi–ii dipole sources were placed inside the microcavity. For Fig. 5biii, a Gaussian beam is placed on the right side of the microcavity to simulate the spatial light input. The frequency domain power monitor obtained the absolute of the electric field distribution. The cross-section of the absolute electric field distribution was calculated by Comsol Multiphysics by a 2D axisymmetric approach. The microcavity was surrounded by a PML. An Eigenfrequency study step was used to compute the field distribution.

### Photoshooting process

Figure 4b was captured by an inverted microscope (Thorlabs Mini Microscopes). To filter the pumping light, we inserted a mirror (with high transmission >99.9% between 1050 nm and 1070 nm and high reflection >99.9% in the range of 780 nm and 830 nm) into the microscope.

### Supplementary information

Supplementary

Exfoliation of YAG crystalline film

## Data Availability

Data underlying the results presented in this paper are available from the corresponding author upon reasonable request. The data that support the findings of this study are available from the corresponding author upon reasonable request.
