# Supplementary material for: Optically pumped Milliwatt Whispering-Gallery microcavity laser
Source: Light Sci Appl. 2023 Sep 12;12:223. doi: 10.1038/s41377-023-01264-6 (PMC10495457; doi:10.1038/s41377-023-01264-6)
Supplement: Supplementary file 1 — Supplementary [file 41377_2023_1264_MOESM1_ESM.docx]

Optically Pumped Milliwatt Whispering-Gallery Microcavity Laser

Huiqi Li, Zhaocong Wang, Lei Wang, Yang Tan^*^ and Feng Chen^*^

School of Physics, State Key Laboratory of Crystal Materials, Shandong University, Shandong, Jinan, 250100, China.

This Supplemental Material is organized as follows. In Section I, we discuss the characteristics of the exfoliated thin crystalline film. In Section II, we make some supplementary remarks on the preparation process. In Section III, we analyze the pump coupling efficiency of eccentric microcavity with different geometric sizes. In Section IV, we compare the experimental parameters of solid-state WGM laser.

**Section I. Carbon-implantation enhanced etching**





**Fig.** S1**:** Transmission Electron Microscope (TEM) images of Nd: YAG, corresponding to range (i) (a), range (ii) (b), range (iii) (c), and exfoliated film (d), respectively.

Fig. S1 displays the Transmission Electron Microscope (TEM) images of Nd: YAG corresponding to different regions shown in Fig. 2. It demonstrates that the lattice damage exists in the electron energy loss region and nuclear energy loss region, while there is no lattice damage exists in the middle region and the exfoliated film.





**Fig.** S2**:** Atomic Force Microscope (AFM) of the exfoliated thin crystalline film. (a) The top facet (boundary of i and ii). (b) The bottom facet (boundary of ii and iii).

We characterize the roughness of the exfoliated thin crystalline film. As exhibited in Fig. S2, the AFM of both sides of the film show that the top facet (the boundary of i and ii) is relatively flat with a roughness of 0.574 nm rms, and the bottom facet (the boundary of ii and iii) is relatively rugged with a roughness of 0.729 nm rms. The roughness of both sides are still in sub-nm level, which ensures that the microcavity can achieve low scattering loss.

**Section II Free-standing crystalline film**

The crystalline Nd: YAG film is exfoliated from the Nd: YAG crystal and transferred to SiO_2_ film by PDMS. Fig. S3a displays the optical image of the crystalline film on PDMS, the mildly homogeneous color indicates a uniform thickness of the crystalline film. Afterward, FIB milling patterned the film into microcavity, eccentric microcavity, and waveguide.





**Fig. S3:** Optical image of (a) exfoliated thin crystalline film (scale bar 30 μm); (b) microcavity (scale bar 10 μm); (c) eccentric microcavity (scale bar 10 μm); (d) waveguide (scale bar 40 μm).

**Section III Optimization of eccentric microcavity**

Fig. S4 shows the coupling efficiency (κ) of the input light into the eccentric microcavity with different positions (d) and radii (r) of the air hole. The highest efficiency is 84.1%, corresponding to r = 2 μm and d =11.2 μm. Therefore, this group of parameters was selected to prepare the eccentric microcavity.





**Fig. S4:** The pump input efficiency(κ) of eccentric microcavity is calculated with different r and d by FDTD solution.

**Section IV Solid-state WGM lasers**

Table S1: Comparison of WGM laser performances

| **Type** | **Doped** | **Diameter**  **(μm)** | **Q-factor** | **Output Power (μW)** | **optical conversion efficiency (%)** | **Ref** |
| --- | --- | --- | --- | --- | --- | --- |
| Silica | Er | 37 | 1.6×10^6^ | 0.13 | 0.65 | 1 |
| Silica | Nd | 37 | 2.6×10^6^ | 0.13 | 0.19 | 2 |
| Silica | Er/Yb | 20 | 3.4×10^6^ | 0.25 | 0.7 | 3 |
| Silica | Er | 121 | 5.2×10^7^ | 0.1 | 3.3×10^-4^ | 4 |
| LNOI | Er | 90 | 1.25×10^6^ | 4×10^-4^ | 6.5×10^-5^ | 5 |
| LNOI | Er | 150 | 1.05×10^5^ | 0.47 | 3.15×10^-3^ | 6 |
| LNOI | Er | 200 | 1.8×10^6^ | 0.14 | 1.91×10^-2^ | 7 |
| LNOI | Yb | 53 | 1.09×10^6^ | 2.5 | 0.53 | 8 |
| LNOI | Yb | 80 | 4×10^6^ | 0.37 | 1.36 | 9 |
| LNOI | Yb | 400 | 1.4×10^6^ | 0.14 | 1.77×10^-3^ | 10 |
| LNOI | Yb | 200 | 1.57×10^5^ | 6.44 | 5.45×10^-2^ | 11 |

**Reference:**

1. Fan, H. et al, Demonstration of an erbium-doped microsphere laser on a silicon chip. Laser Physics Letters **10**, 105809 (2013).
2. Ding, Y. et al, Ultralow-threshold neodymium-doped microsphere lasers on a silicon chip. Optics Communications **395**, 51-54 (2017).
3. Hu, Y. Q. et al, Demonstration of Yb^3+^-doped and Er^3+^/Yb^3+^-codoped on-chip microsphere lasers. Optics Express **29**, 25663-25674 (2021).
4. Zhu, S. et al, All-Optical Tunable Microlaser Based on an Ultrahigh-Q Erbium-Doped Hybrid Microbottle Cavity. ACS Photonics **5**, 3794−3800 (2018).
5. Luo, Q. et al, Microdisk lasers on an erbium-doped lithium-niobite chip. Science China Physics, Mechanics & Astronomy **64**, 234263 (2021).
6. Liu, Y. A. et al, On-chip erbium-doped lithium niobate microcavity laser. Science China Physics, Mechanics & Astronomy **64**, 234262 (2021).
7. Wang, Z. et al, On-chip tunable microdisk laser fabricated on Er^3+^-doped lithium niobate on insulator. Optics Letters **46**, 380-383 (2021).
8. Zhou, Y. et al, On-chip microdisk laser on Yb^3+^-doped thin-film lithium niobate. Optics Letters **46**, 5651-5654 (2021).
9. Luo, Q. et al, On-chip ytterbium-doped lithium niobate microdisk lasers with high conversion efficiency. Optics Letters **47**, 854-857 (2022).
10. Ma, Y. et al, Monolithic Yb^3+^-doped thin film lithium niobate microring laser fabricated by photolithography assisted chemo-mechanical etching technology. Journal of the Optical Society of America B **40**, D1–D4 (2023).
11. Luo, Q. et al, Integrated ytterbium-doped lithium niobate microring lasers. Optics Letters **47**, 1427-1430 (2022).
